# Imageability: now you see it again (albeit in a different form)

**DOI:** 10.3389/fpsyg.2014.00279

**Published:** 2014-04-03

**Authors:** Sara Dellantonio, Remo Job, Claudio Mulatti

**Affiliations:** ^1^Department of Psychology and Cognitive Science, University of TrentoRovereto (Trento), Italy; ^2^Department of Developmental Psychology and Socialisation, University of PaduaPadova, Italy

**Keywords:** emotions, context effects, imageability, lexical access, co-occurrence statistics

Imageability is a complex and controversial measure as was already noted by Paivio et al. (1968) when they first introduced the term. According to these authors imageability should describe the ease/difficulty with which “words arouse a sensory experience” (p. 4) and therefore should closely correlate with concreteness. Since concrete objects are experienced through the senses, the words that denote them should easily re-evoke that sensory experience: words high in concreteness should also be high in imageability and vice versa. However, Paivio et al. also noted an interesting asymmetry: some terms with low concreteness ratings (i.e., abstract terms) had high imageability ratings. The peculiarity of these words is that they have strong emotional connotations (Paivio et al., 1968, p. 7). (They also noted a few terms with high concreteness and low imageability ratings such as e.g., *astrolab*, but these are easy to explain: people know that they denote concrete objects, but they do not know what they look like).

Westbury et al. (2013) aim is mainly pragmatic, and consists in “estimating imageability using algorithmic methods grounded in empirical measures,” and we are quite sympathetic to this endeavor. Nevertheless, the measures these authors rely on clearly imply a theoretical account able to deal with the anomalies of this construct. Their strategy is to distinguish two aspects of imageability: (a) the relationship between imageability and context, and (b) the relationship between imageability and emotionality. The line of reasoning they adopt to develop their method is the following.

The first measure introduced is “density of the context,” a measure derived from *Context Availability Theory* which maintains that the difference between concrete and abstract words can be reduced to the fact that it is easier (faster) to describe the properties of concrete than of abstract words when they are presented without context. “Density of the context” is a measure which reflects how low imageability words—like low concreteness words—depend more on contextual information than high imageability (and high concreteness) words. In this sense words with high imageability ratings are considered to behave analogously to words with high concreteness playing a more important role when context is less dense.

However, as e.g., Prinz (2002) notes, the context effects derived from Context Availability Theory are also compatible with the predictions made by its most important competitor, i.e., Paivio's *Dual Coding Theory* (1986, 2007), which proposes an explanation of the abstract/concrete difference that is radically different from a psychological perspective: “In the dual-code model, abstract word comprehension relies on verbal information. As a result, processing speed for abstract words should increase when more verbal information is provided. The dual-code model also predicts that there will be no comparable improvement in performance on concrete words because performance is already near ceiling levels when concrete words are presented in isolation” (Prinz, 2002, p. 132). If the two accounts make the same prediction, then the results of Westbury and colleagues do not necessarily favor the Context Availability Theory over the Dual Coding Theory.

Thus, the reason why the density of the context is a good predictor of imageability does not necessarily relate to the fact that the difference between concrete/imageable vs. abstract words can be reduced to differences in the way these words depend on context and the possibility that imageability may rely on semantic properties cannot be ruled out. In fact, the density of the context would also be a good measure of imageability if the difference between concrete/imageable vs. abstract depended on how the semantic system is structured, as Paivio suggests, since the way words are represented influences whether they rely strongly or weakly on context.

Second, imageability is related to abstractness and is described on the basis of the theoretical account largely developed by Altarriba et al. (1999) and Kousta et al. (2011) who maintain that “affective information (emotional association […]) is more important for abstract than concrete concepts.” From these studies Westbury and collaborators derive the second predictor of imageability, which they call “emotional valence,” and which they claim can account for the asymmetry already identified by Paivio and collaborators: For some words characterized by strong emotional connotations imageability is significantly higher than concreteness.

Taken together, these two measures describe imageability in the way Paivio et al. did when the construct was originally introduced. In fact, they reproduce the intrinsic ambiguity of the words with high or relatively high imageability ratings which, if they also have high concreteness ratings behave like concrete words, but if they have low concreteness ratings are of high emotional valence. Notably, these observations challenge Westbury and collaborators' assumption that “imageability judgments are correlated with factors that have nothing to do with evoked sensation […].” On the contrary, one could argue that the factors they identify support the hypothesis that imageability measures evoke sensations; however, they support this claim in a slightly different version also suggested by Paivio in his later works. Words with high imageability and high concreteness ratings evoke sensations connected to the perception of the objects they denote, while words with high imageability and low concreteness ratings evoke sensations connected to affective arousal (Paivio, 1986, 2007).

## Conflict of interest statement

The authors declare that the research was conducted in the absence of any commercial or financial relationships that could be construed as a potential conflict of interest.
